# Enhancing HR Frequency for Precise Genome Editing in Plants

**DOI:** 10.3389/fpls.2022.883421

**Published:** 2022-05-03

**Authors:** Hao Chen, Matthew Neubauer, Jack P. Wang

**Affiliations:** ^1^Department of Plant and Microbial Biology, Program in Genetics, North Carolina State University, Raleigh, NC, United States; ^2^College of Forestry, Shandong Agricultural University, Tai’an, China; ^3^Department of Forestry and Environmental Resources, Forest Biotechnology Group, North Carolina State University, Raleigh, NC, United States; ^4^State Key Laboratory of Tree Genetics and Breeding, Northeast Forestry University, Harbin, China

**Keywords:** homologous recombination, homology-directed repair, gene targeting, donor template, programmable nucleases

## Abstract

Gene-editing tools, such as Zinc-fingers, TALENs, and CRISPR-Cas, have fostered a new frontier in the genetic improvement of plants across the tree of life. In eukaryotes, genome editing occurs primarily through two DNA repair pathways: non-homologous end joining (NHEJ) and homologous recombination (HR). NHEJ is the primary mechanism in higher plants, but it is unpredictable and often results in undesired mutations, frameshift insertions, and deletions. Homology-directed repair (HDR), which proceeds through HR, is typically the preferred editing method by genetic engineers. HR-mediated gene editing can enable error-free editing by incorporating a sequence provided by a donor template. However, the low frequency of native HR in plants is a barrier to attaining efficient plant genome engineering. This review summarizes various strategies implemented to increase the frequency of HDR in plant cells. Such strategies include methods for targeting double-strand DNA breaks, optimizing donor sequences, altering plant DNA repair machinery, and environmental factors shown to influence HR frequency in plants. Through the use and further refinement of these methods, HR-based gene editing may one day be commonplace in plants, as it is in other systems.

## Homologous Recombination in Plants: An Ideal Genome Engineering Tool With Low Efficiency

**Figure 1:**
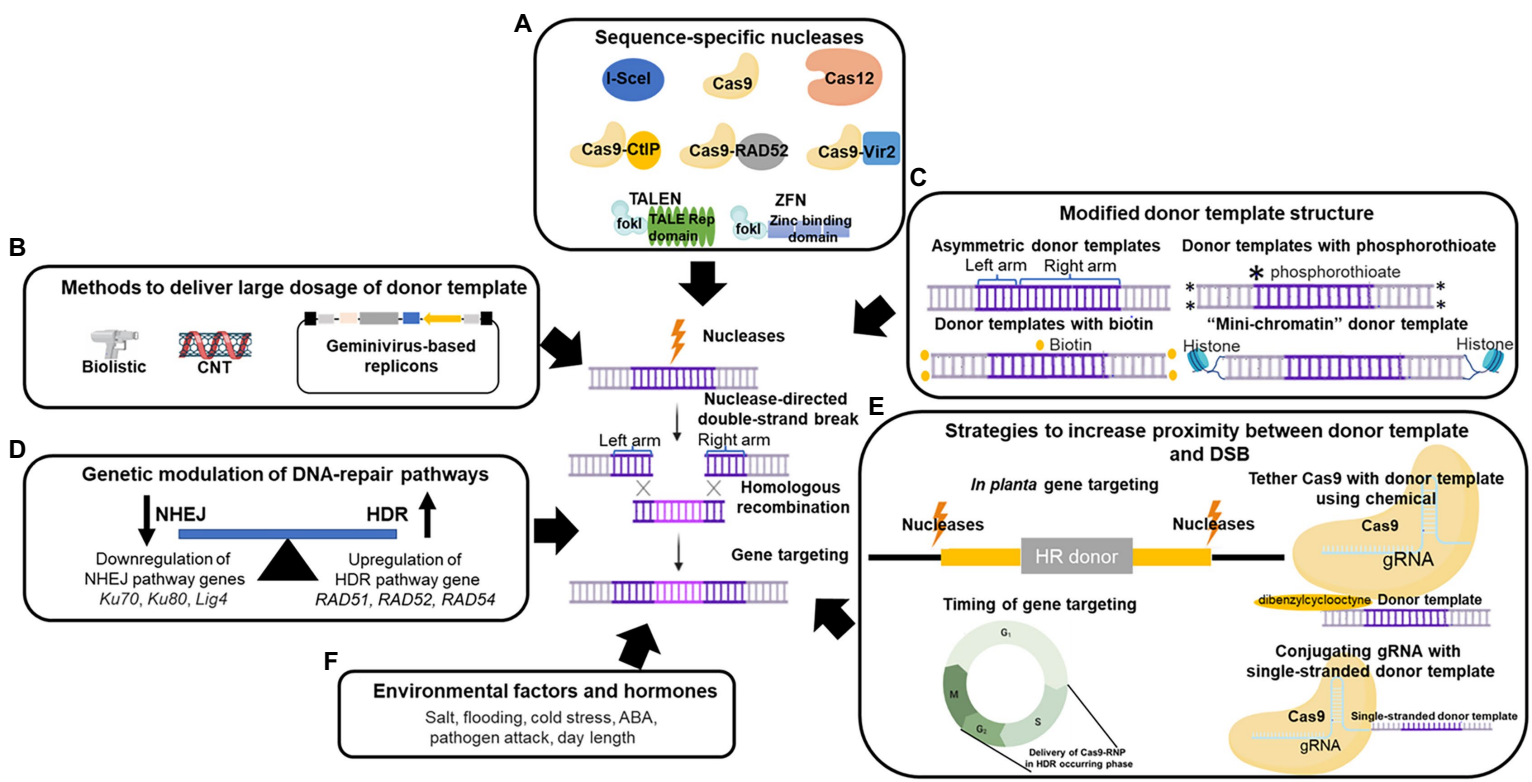
Approaches for enhancing homologous recombination (HR) and gene targeting (GT) frequency in plants. **(A)** Sequence-specific nucleases can induce double-strand breaks (DSBs) at target loci. These nucleases include I-SceI, TALENs (TALE repetitive domain with FokI), ZFNs (Zinc-finger binding domain with FokI), Cas9, Cas12a, and Cas9 fusion proteins (Cas9-CtIP, Cas9-RAD52, and Cas9-Vir2). **(B)** Methods that can increase the dosage of donor templates in plants, encompassing biolistic, carbon nanotubes (CNTs), and geminivirus-based replicons (GVRs). **(C)** Modifications of donor template structure that result in higher HR efficiency. Altering the donor template symmetricity, labeling the 5′ or 3′ end of the donor template using phosphorothioate linkages or biotin, and attaching the donor template with histones as “mini-chromatin” can improve GT efficiency in plant cells. **(D)** Improved GT efficiency by genetic manipulation of the NHEJ and HR pathways. Knockout mutations in NHEJ pathway genes *Ku70*, *Ku80*, and *Lig4,* and the overexpression of HDR pathway genes *RAD51*, *RAD52*, and *RAD54* can enhance HR efficiency in plants. **(E)** Strategies for bringing donor templates to target loci. These strategies include *in planta* gene targeting, delivery of the donor templates to the DSB during cell cycle phases when HDR occurs, conjugating the gRNA with the donor template, and attaching the donor template with the nuclease. **(F)** Environmental factors and hormones that alter HR efficiency. *Designates phosphorothioate modification at the end in donor template.

Homologous recombination (HR) is a complex process whereby DNA segments that share significant sequence homology are exchanged. In some organisms, such as bacteria and yeast, DNA integration occurs primarily through HR. When double-stranded DNA breaks at a given locus, HR can accurately transfer a donor sequence that contains flanking regions of homology into the targeted locus (San Filippo et al., 2008). Using HR-based gene editing, scientists have successfully performed targeted sequence insertions, replacements, and point mutations by exchanging the original sequence with designed donor sequences (Hoshijima et al., 2016; Ghosh et al., 2021). The highly specific genome edits enabled by HR have led to widespread use of the technique in yeast, bacteria, and vertebrates (Bernardi and Wendland, 2020). HR-mediated genome editing in plants, generally referred to as gene targeting (GT), was first achieved by Paszkowski et al. (1988) in tobacco cells. This work focused on restoring a defective kanamycin-resistance gene using plasmids bearing the missing sequence through homologous recombination in protoplasts (Paszkowski et al., 1988). Although this approach was successful, the observed GT frequency in tobacco protoplasts was only 0.5–4.2 × 10^−4^, consistent with the natural rate of HR-based repair in higher plants, which ranges from 10^−3^ to 10^−6^ (Paszkowski et al., 1988; Mengiste and Paszkowski, 1999; Terada et al., 2002). To this day, the low efficiency of HR in higher plants is a major barrier to the wider application of GT in crop genome engineering and plant genetics research (Puchta and Fauser, 2013; Barakate et al., 2020; Miller et al., 2021). Here, we review work aimed at improving the efficiency of HR-based plant genome engineering and discuss possible strategies for maximizing GT efficiency in plants (Figure 1).

## Programmable Nucleases and Nickases: Molecular Scissors to Enhance HR Frequency in Plant Cells

Puchta et al. (1993) performed the first experiments to induce site-specific breaks in a plant genome using the homing endonuclease *Saccharomyces cerevisiae* endonuclease I-SceI (Puchta et al., 1993). By recognizing a non-palindromic 18mer recognition sequence present into the genome, I-SceI induced double-strand breaks (DSBs) at the sequence-specific locus (Perrin et al., 1993; Puchta et al., 1993; Moure et al., 2003). The DSB created by transiently expressed I-SceI was successfully repaired by HR using sequences bearing homology to the target locus provided by an exogenous T-DNA in *Nicotiana plumbaginifolia* protoplasts (Puchta et al., 1993, 1996). Notably, introducing DSBs at the I-SceI target locus and supplying the cell with homologous repair fragments increased the frequency of HR by around 100-fold relative to the naturally occurring rate in tobacco cells, of roughly 10^−5^ to 10^−3^ (Puchta et al., 1996; Puchta, 2005). DSBs induced by I-SceI resulted in high frequency HDR not only in the dicot plant tobacco, but in the monocot species maize and barley also resulted in high frequency of HDR (D’Halluin et al., 2008; Ayar et al., 2013; Watanabe et al., 2016). A major drawback to this approach is that it requires the presence of an I-SceI recognition site at the targeted locus. Fortunately, multiple nucleases with programmable and sequence-specific recognition sites have been developed to induce DSBs at specific loci.

Programmable nucleases are usually created by combining DNA-binding domains that recognize specific genetic loci with a nuclease domain that nicks the DNA (Gupta and Musunuru, 2014; Kim and Kim, 2014; Chandrasegaran and Carroll, 2016). This was first accomplished by a combination of a highly variable class of zinc-finger transcription factors and a cleavage domain of the restriction enzyme FokI (Kim et al., 1996; Smith et al., 2000). Zinc-finger nucleases (ZFN) were used to create site-specific DSBs, which greatly promotes the occurrence of HR. For instance, the introduction of ZFN-mediated DSBs resulted in HR-mediated repair of approximately 20% of *GUS:NPTII* reporter genes in tobacco protoplasts (Wright et al., 2005), while the remainder was modified by a combination of HR and NHEJ. In later work, the Voytas group successfully used ZFN-mediated gene targeting to engineer the endogenous tobacco herbicide-resistance genes (*ALS SuRA and SuRB*), where the HR frequency ranged from 0.2 to 4% for these endogenous genes (Townsend et al., 2009). This approach has not only been successful in dicots, but also in monocot crops. In maize, ZFN-mediated GT was used to restore *IPK* function, resulting in the transgenics with an herbicide-resistant phenotype and altered inositol phosphate content in seeds (Shukla et al., 2009). Like ZFNs, transcription activator-like effector nucleases (TALENs), coupled with HR, have been used in plant genome engineering (Baltes et al., 2014; Čermák et al., 2015). TALENs are composed of a TAL effector DNA-binding domain and a FokI nuclease domain which act as dimers for recognizing and cutting the target sites (Miller et al., 2011). GT experiments using TALENs in tobacco protoplasts demonstrated that it is possible to introduce a 6 bp modification to the *ALS* gene with GT frequencies of up to 4% (Zhang et al., 2013), which is comparable to ZFN-mediated GT frequency (Zhang et al., 2010). With enhancing GT frequency, these programmable nucleases were also used for precise stacking of multiple crop traits in a single locus through HR, thus generating transgenic plants with multiple linked advantageous traits (Ainley et al., 2013; Kumar et al., 2015; Demorest et al., 2016).

The clustered regularly interspaced short palindromic repeat (CRISPR)-Cas9 system has proven to be another useful tool for introducing DSBs into plant genomes (Wiedenheft et al., 2012; Shan et al., 2013; Zhu et al., 2020). HR-mediated GT experiments utilizing CRISPR-Cas9 have been performed in numerous plant species, such as Arabidopsis (Li et al., 2013; Hahn et al., 2018), tobacco (Li et al., 2013), maize (Shi et al., 2017; Agarwal et al., 2018), rice (Li et al., 2016; Romero and Gatica-Arias, 2019; Van Vu et al., 2019), and tomato (Dahan-Meir et al., 2018; Van Vu et al., 2020). Unlike ZFNs and TALENs, which use FokI to create DSBs at the target sequence, the CRISPR-Cas9 approach takes advantage of a guide RNA to direct the Cas9 nuclease to the target site (Wiedenheft et al., 2012). The use of *Streptococcus pyogenes* Cas9 (SpCas9) by Li et al. (2013) for HR-mediated GT resulted in successful GT in 9% of tobacco protoplasts; a result comparable to the use of ZFN- and TALEN-mediated GT. Despite this success, GT experiments using CRISPR-Cas9 have not demonstrated high efficiency in all tested species. For example, this approach did not enable HR-mediated GT in Arabidopsis protoplasts (Li et al., 2013). Although HDR was achieved using transgenic Arabidopsis lines with high levels of Cas9 expression in the germline (Miki et al., 2018), attempts at improving the low efficiency of HR-mediated GT in Arabidopsis have largely been unsuccessful (Shan et al., 2018). In Arabidopsis, the nickase Cas9 was found to induce HR at a similar frequency as the nuclease Cas9 or I–SceI (Fauser et al., 2014). In another experiment, Cas9 nickase was used in place of the Cas9 nuclease, and regenerated Arabidopsis harboring HR-mediated repair of the *glabrous1* gene was detected at a frequency of only 0.12% (Hahn et al., 2018). These Arabidopsis experiments that used different variants of Cas9 raised the question of whether different nucleases or nickases may alter the efficiency of HR *via* their specific strand-breaking mechanism, and whether specific features of these enzymes may enhance HR-mediated GT in Arabidopsis.

Another widely used Cas protein, Cas12a (Bernabé-Orts et al., 2019), was tested for its ability to enhance HR-mediated GT efficiency in Arabidopsis, tobacco, and tomato. The use of *Lachnospiraceae bacterium* Cas12a (LbCas12a) resulted in a greater HR-mediated GT frequency than that observed with Cas9 in Arabidopsis or tomato (Wolter and Puchta, 2019; Van Vu et al., 2020). Overall, the frequency of GT observed using LbCas12a is around 50% higher than that achieved using Cas9 in Arabidopsis, and 3-fold higher than that achieved with Cas9 in tomatoes (Van Vu et al., 2020). GT based on Cas12a may be more efficient than Cas9 due to two unique ways Cas12a processes DNA. One difference between the two enzymes is that when Cas12a cuts DNA, it produces staggered 5′ overhangs, while Cas9 produces blunt ends (Zetsche et al., 2015; Bothmer et al., 2017; Swarts and Jinek, 2018; Huang and Puchta, 2019). Cohesive ends generated by these staggered cuts may facilitate the invasion of the donor template into the targeted DNA, followed by HR-based double-strand DNA repair (Puchta, 1998; Zetsche et al., 2015; Huang and Puchta, 2019). Another unique feature of Cas12a is that the DSBs it creates are located outside of the targeted genomic region recognized by the guide RNA (Zetsche et al., 2015), which preserves the sequence of the target locus until HR-mediated GT has occurred. If Cas12a is proven to enable greater GT efficiency in additional plant species, it is likely an ideal programmable tool for GT in plants.

Fusion of Cas proteins with HR-mediated repair pathway proteins has been shown to enhance HR efficiency in mammalian species. Cas9 proteins were fused with either yeast RAD52, which promotes strand invasion (Shao et al., 2017), or human CtIP, which is involved in DNA resection at the early steps of homologous recombination (Charpentier et al., 2018). These protein fusions resulted in a more than 2-fold increase in HR efficiency in mammalian cells compared to the native Cas9 (Shao et al., 2017; Charpentier et al., 2018). Given that RAD52 and CtIP functions are conserved in HR-mediated repair pathways across mammals and plants (Manova and Gruszka, 2015), it is worth testing whether Cas9 fusions with HDR pathway proteins also improve HR efficiency in plants.

## Donor Template Structure: What Donor Template is Ideal for Enhancing HR Frequency in Plant Cells?

One strategy for optimizing HR frequency is to determine the optimal type or structure of the donor template. The first exogenous donor template used for HR-mediated GT was double-stranded DNA (dsDNA), delivered as a plasmid in tobacco protoplasts. Using the same tobacco protoplast system, HR-mediated GT was achieved using a T-DNA that carried the donor dsDNA template. The HR frequency observed between the T-DNA and the target locus was comparable to that between plasmids and the target locus (Offringa et al., 1990). In subsequent experiments, DNA oligos and DNA–RNA hybrid oligos containing regions homologous to the target sequence were used as donor templates for HR in various plant species, including maize, tobacco, rice, and wheat (Beetham et al., 1999; Zhu et al., 1999, 2000; Okuzaki and Toriyama, 2004; Dong et al., 2006). The use of chimeric oligonucleotides increased the efficiency of GT to ~10^−4^ in these species, which is higher than the HR frequency obtained using a single donor dsDNA template (Beetham et al., 1999; Zhu et al., 1999, 2000; Okuzaki and Toriyama, 2004; Dong et al., 2006). Additionally, single-stranded DNA (ssDNA) has been used as a donor template in plants (Bilang et al., 1992; Svitashev et al., 2015), but with no obvious improvement in HR-mediated GT efficiency compared to dsDNA. This observation in plants is surprising, considering that ssDNA was superior to dsDNA as a template for HDR in zebrafish (Bai et al., 2020). ssRNA has also been used as a donor template for GT in rice. However, the observed HR frequency was significantly lower than that observed when ssDNA was used as the donor template (Li et al., 2019).

Recently, efforts to further modulate donor template structure have been made to enhance HR frequency in mammalian species. Several strategies could significantly enhance HR-mediated GT, including modifying the lengths and ratios of the homologous and non-homologous parts of the donor templates (Baker et al., 2017; Zhang et al., 2017), altering the donor template sequence symmetricity (Richardson et al., 2016; Moreno-Mateos et al., 2017), labeling the 5′ or 3′ end of donor templates using phosphorothioate linkages (Renaud et al., 2016) or biotin (Gutierrez-Triana et al., 2018), and attaching the donor template with histones as “mini-chromatin” (Cruz-Becerra and Kadonaga, 2020). Some of the above strategies have been used in plant genome engineering, although the mechanisms through which these strategies improve HR frequency are poorly understood. For example, in rice, chemical modification of the donor template using phosphorothioate linkages at the 5′ and 3′ ends improved the HR-mediated GT frequency compared to donor templates lacking such modifications (Ali et al., 2020). This improvement may be due to the end modifications protecting the donor templates from degradation (Ali et al., 2020), which are also observed in NHEJ-mediated repair in rice (Li et al., 2019). Lu et al., also observed higher HDR efficiencies when tandemly repeated sequences are present near DSBs and then developed a tandem repeat-HDR strategy (TR-HDR) to achieve targeted GT. This TR-HDR was successfully used to introduce in-locus tags, with editing efficiencies ranged from 3.4 to 11.4% in rice (Lu et al., 2020).

## Improving HR Frequency by Increasing Donor Template Dosage

Protoplasts are a useful tool for implementing and monitoring HR-based genome editing (Beard et al., 2021; Wright et al., 2005; Townsend et al., 2009; Puchta and Fauser, 2013; Zhu et al., 2020; Hsu et al., 2021). Working with protoplasts enables the convenient and efficient delivery of large quantities of donor template as well as DNA that encodes for sequence-specific nucleases (Sant’Ana et al., 2020; Nicolia et al., 2021; Lin et al., 2022). It has been hypothesized that efficient delivery of large amounts of donor template results in increased HR-mediated GT efficiency (Baltes et al., 2014). To address this question, Baltes et al. (2014) developed geminivirus-based replicons (GVR) to deliver and produce high levels of donor templates to plant cells (Baltes et al., 2014). The GVRs used sequences derived from the Bean Yellow Dwarf Virus (BeYDV), including the short intergenic region (SIR), two copies of the long intergenic region (LIR) flanking the replicon cargo, and the Rep/RepA replicase expression cassette for replicon formation and amplification. The GVR, which contained a nuclease expression cassette and donor template, was delivered as a T-DNA to tobacco epidermal cells using agrobacterium-mediated transformation. Following transformation, rolling circle replication of the replicon initiates at the LIR sites, resulting in the assembly of the circularized replicon (Baltes et al., 2014). The circularized replicon can then undergo further rounds of replication, thereby generating large amounts of the donor templates (Baltes et al., 2014). In a transgenic *N. tabacum* reporter line, the GVR approach enhanced GT efficiency by one to two orders of magnitude relative to traditional T-DNA delivery of the repair template (Baltes et al., 2014). Further studies using this approach also demonstrated that including the repair template within the replicon enhanced GT efficiency, whereas including the nuclease within the replicon had a negligible effect on GT efficiency (Baltes et al., 2014; Wang et al., 2017).

Geminivirus replicon (GVR)-based GT has been employed in various plant species, such as tomato, potato, tobacco, and rice (Baltes et al., 2014; Čermák et al., 2015; Butler et al., 2016; Gil-Humanes et al., 2017; Wang et al., 2017). Notably, a BeYDV-based GVR system was employed to enhance donor template delivery in tomato. This approach proved to be highly efficient, resulting in the precise repair of *crtiso* mutants at a frequency of 25% (Dahan-Meir et al., 2018). A Wheat Dwarf Virus (WDV)-based GVR system was employed for GT in rice, where the *ACT1* and *GST* loci were successfully modified in up 19.4% of instances (Wang et al., 2017). Despite the successful use of GVRs for improving GT in these species, GVR-based approaches have failed in Arabidopsis (de Pater et al., 2018; Hahn et al., 2018; Shan et al., 2018). Hahn et al. observed that only 3 in thousands of regenerated Arabidopsis plants underwent successful HR-mediated GT, whereas no plants were successfully regenerated following GVR-based editing (Hahn et al., 2018). Another report failed to recover heritable GT events in Arabidopsis using a BeYDV GVR, despite a high HR frequency observed among transformed plants (Shan et al., 2018). This problem is not limited to Arabidopsis. The regeneration of plants edited using GVR-based methods has been an issue in multiple species (Gil-Humanes et al., 2017; Hummel et al., 2018). For example, despite a successful insertion of fluorescent marker genes into wheat cells and scutella using a WDV-based replicon approach, the regeneration of edited plants is not reported (Gil-Humanes et al., 2017). Based on these observations in wheat and Arabidopsis, it was hypothesized that the geminivirus-based replicons and replicase machinery might negatively impact plant regeneration and growth (Gil-Humanes et al., 2017). This hypothesis is supported by experiments in cassava employing GVRs (Hummel et al., 2018). Many of the regenerated cassava plants edited using BeYDV replicons displayed stunted growth and leaf chlorosis, which are common disease symptoms associated with viral infection (Lei et al., 2017; Hummel et al., 2018). Given these limitations, alternative methods or modifications to existing GVR-based replicon systems may be necessary to employ replicon-based genome editing approaches in plant species. Additionally, agrobacterium-based delivery of morphogenic or developmental regulators (WUS, MP, and BBM) together with the gene-editing machineries also improved the efficiency of HR-mediated GT (Jones et al., 2019; Maher et al., 2020; Peterson et al., 2021), by increasing the number of transgenics recovered and screening accuracy. However, the effects of these regulators on HR frequency have not been investigated. Another approach to delivering large amounts of donor template is biolistic delivery (Ozyigit and Kurtoglu, 2020), which utilizes microparticles coated with DNA. Once these particles are introduced into plant cells, the DNA dissociates from the particles and can be transiently expressed or integrated into the host genome. Relative to *Agrobacterium*-mediated delivery of a donor template, biolistic approaches allow for greater control over the amount of donor template delivered by coating defined quantities of the template into particles, which could positively impact GT frequency. In maize, successful GT events were observed in 4.1% of cases when a biolistic-based method was used. In contrast, no successful GT was observed when the donor template was instead delivered by *Agrobacterium*-mediated transformation, despite the same nucleases and donor template being used (Svitashev et al., 2015). Additionally, biolistic-based approaches enable genome editing without integration of the editing machinery into the genome by directly delivering DNA-RNA hybrids, RNA, DNA-protein complexes, RNA-protein complexes, or chemically modified nucleotides to plant cells (Zhang et al., 2016; Ozyigit and Kurtoglu, 2020). Given that modifications to donor template structure or composition can improve HR-mediated GT frequency, biolistic approaches for delivering donor templates provide an advantage over *Agrobacterium*-based approaches for HR-mediated GT. Despite these advantages, work in rice and maize has demonstrated that biolistic methods often induce unintended sequence disruptions to the host genome, such as additional DNA breaks and shearing (Liu et al., 2019). These off-target effects are a significant drawback to using biolistic-based methods, especially when a major motivation for using HR-mediated GT for genome engineering is its ability to modify the target precisely and accurately. Therefore, another delivery method capable of shuttling large amounts of donor template that does not negatively impact genomic integrity or the ability to regenerate transgenic plants is highly desirable for improved GT in plants.

Carbon-based nanoparticles have the potential to be used for genome editing by enabling the delivery of DNA that can be transiently expressed or used as repair templates for HDR (Demirer et al., 2019a,b; Ahmar et al., 2021). Carbon nanotubes (CNTs) have been utilized to successfully deliver DNA for expression in plant cells. CNT-based approaches could enable species- and tissue-independent passive delivery of DNA, RNA, and proteins. DNA delivered using CNTs could be expressed at high levels, and no genomic damage has been reported (Cunningham et al., 2018; Demirer et al., 2019a,b; Ahmar et al., 2021). Like biolistic-based approaches, CNT-mediated delivery of DNA to plant cells should enable some degree of control over the amount of donor template delivered to plant cells. CNT-based delivery of nuclease-encoding plasmids may also enable highly efficient genetic editing without transgene integration. Transient expression of gene-editing reagents has been shown to result in lower off-target editing and toxicity relative to methods that rely on genome integration and stable expression. CNT-based repair template delivery may prove superior to biolistic-based delivery approaches by avoiding the genomic damage induced by biolistic delivery (Ahmar et al., 2021). Given these advantages, CNT-based approaches may prove useful for HR-mediated GT for the delivery of genome editing machinery as well as donor templates. Additionally, non-Agrobacterium species have been used to enhance HDR in plants (Kumar et al., 2022). O. haywardense was used to deliver CRISPR-Cas9 components and donor template into soybean. T0 plants were regenerated 6-8 weeks after transformation, with observed GT efficiency higher than that resulting from particle bombardment-mediated delivery (Kumar et al., 2022).

## Face-to-Face: Bringing the Donor Template and Target Locus Together Improves HR Frequency

Given that high copies of donor templates can positively impact HR frequency, approaches that position the donor templates near the target locus may have a similar effect. In plants, one such approach, *in planta* gene targeting (*in planta* GT), has been successfully used to enhance HR-mediated GT (Fauser et al., 2012). The principle behind *in planta* GT is to increase the spatial and temporal availability of donor template sequences to the DSB position, thereby enhancing the frequency of HR-mediated GT. The *in planta* GT method involves the integration of the donor template and nuclease-encoding sequences into the same chromosome bearing the target site. Subsequent expression of the nucleases resulted in the excision and release of the donor template and the induction of dsDNA breaks at the target locus (Fauser et al., 2012; Wolter and Puchta, 2019). *In planta* GT enables the donor template to be produced simultaneously and near the DSB generated by the nuclease. Using I-SceI-based *in planta* GT in Arabidopsis, the frequency of HR-mediated GT reached 10^−2^ (Fauser et al., 2012), denoting one GT event per 100 seeds could be recovered. The recent *in planta* GT experiment in Arabidopsis used LbCas12a as the nuclease, which further improved the frequency of HR-mediated GT (Wolter and Puchta, 2019). *In planta* gene targeting were also successfully applied in crop species, such as maize (Barone et al., 2020; Peterson et al., 2021), and barley (Lawrenson et al., 2021).

Similar attempts to improve HR frequency by increasing the spatial and temporal availability of donor sequences have been made in yeast and animals. These attempts included the delivery of nuclease and donor sequences to the target site during S/G2 phase when HDR occurs (Lin et al., 2014), the conjugation of single-stranded donor templates to the gRNA (Lee et al., 2017), the use of retrons (Sharon et al., 2018), the attachment of the donor template directly to the nuclease or by DNA aptamer (Ling et al., 2020), attachment of nucleases to donor template-interacting proteins (Aird et al., 2018; Savic et al., 2018) or proteins that localize near the target site (Roy et al., 2018). These attempts at co-localizing donor templates with target loci significantly increased HR-mediated GT frequency 3- to 30-fold. Most of the above approaches may similarly improve HR frequency in plants but have yet to be applied. To our knowledge, the only attempts at bringing the donor sequence and target loci together for improved gene targeting were performed in rice (Ali et al., 2020). Ali et al. (2020) fused Cas9 with a VirD2 relaxase to bring the donor template close to the targeted DSB site. The Cas9-VirD2 fusion protein enhanced the efficiency of HDR repair by more than 4-fold compared to Cas9 alone and enabled precise modification of *ACETOLACTATE SYNTHASE* (*OsALS*) allele, the *OsCCD7* gene, and to make an in-frame epitope tag fusion at *OsHDT* for generating herbicide-resistant and trait-modified rice (Ali et al., 2020).

## The DNA Repair Seesaw: Balancing DNA Repair Machineries to Enhance HR Frequency

When DSBs occur, plant cells employ HR to accurately repair the damaged locus or NHEJ to repair the template imprecisely (Johnson and Jasin, 2001; Manova and Gruszka, 2015). In higher plants, the naturally occurring rate of NHEJ is much higher than that of HR, resulting in an imbalance between precise repair (HR) and imprecise repair (NHEJ; Schmidt et al., 2019). This competition between HR and NHEJ in repairing DSBs has been observed in many species, including yeast, animals, and plants (Shrivastav et al., 2008; Kass and Jasin, 2010; Gratz et al., 2014; Manova and Gruszka, 2015; Schmidt et al., 2019). In animal species, knockout mutations in NHEJ pathway genes have been shown to repress NHEJ-mediated repair and enhance HR frequency (Pierce et al., 2001). Given the advantages of HR-mediated GT over NHEJ-mediated repair for genome engineering, many studies have investigated the effects of suppressing the NHEJ pathway to enhance HR using chemical and genetic approaches (Beumer et al., 2008; Maruyama et al., 2015; Nakanishi et al., 2015; Robert et al., 2015; Weber et al., 2015; Devkota, 2018). These studies have primarily focused on NHEJ regulators, including the ku70/ku80 heterodimer, DNA-protein kinase catalytic subunit (DNA-PKcs), and DNA ligase IV (Davis and Chen, 2013). Since no DNA-PKcs kinase has been identified in plants (Manova and Gruszka, 2015), plant biologists have primarily studied the HR frequency in *ku70/80* and *DNA ligase IV* mutants. Studies in Arabidopsis have demonstrated a 5- to 16-fold increase in HR-mediated GT in *ku70* mutants and a 3- to 4-fold increase in GT in *lig4* mutants (Qi et al., 2013). In contrast, the intrachromosomal HR frequency in *ku80* is close to that in wildtype plants (Gallego et al., 2003). A similar effect was observed in rice, where knocking-down NHEJ regulators, including *OsKu70*, *OsKu80*, and *OsLig4,* increased the frequency of HR (Nishizawa-Yokoi et al., 2012). Subsequent work showed that knockout mutations in *OsLig4* could enhance HR-mediated GT in rice, enabling greater HR-mediated replacement of *acetolactate synthase* (*ALS*; Endo et al., 2016). Consistent results were demonstrated in another experiment in which knocking-down *ku70/80* or *Lig4* enhanced the efficiency of HR in rice (Nishizawa-Yokoi et al., 2012). Given that *ku70/80* and *Lig4* mutants display growth defects (Nishizawa-Yokoi et al., 2012; Qi et al., 2013), an inducible system in which the expression of *ku70/80* or *Lig4* could be controlled following the delivery of genome editing machinery and donor template could improve HR-mediated GT frequency in plants.

Effort has also been made in analyzing the effects of modifying the homology-direct repair (HDR) pathway in plants to improve HR-mediated GT (Lieberman-Lazarovich and Levy, 2011; Pradillo et al., 2014; Steinert et al., 2016; Hernandez Sanchez-Rebato et al., 2021). In yeast, the core components of the HR machinery are RAD51, RAD52, and RAD54 (Li and Heyer, 2008). In Arabidopsis, five RAD51 homologs have been identified as: *AtRAD51B*, *AtRAD51C*, *AtRAD51D*, *AtXRCC2,* and *AtXRCC3* (Osakabe et al., 2005). Plants with knockout mutations in *AtRAD51B*, *AtRad51C*, *AtRAD51D*, and *AtXRCC2* were found to have reduced HR frequencies (Abe et al., 2005; Serra et al., 2013). Two RAD52 homologs, *AtRAD52-1* and *AtRAD52-2*, have been identified in Arabidopsis (Samach et al., 2011). Overexpression of nuclear-localized *AtRAD52-1A* enhanced the HR-mediated GT frequency in Arabidopsis only when the target gene was also targeted by RNAi (Samach et al., 2018), suggesting that the siRNA pathway may affect HR machinery. This hypothesis is supported by the observation that all tested Arabidopsis siRNA biogenesis defective mutants (*Dicer-like 2* (*DCL2*), *DCL3,* and *DCL4, RNA-dependent RNA polymerase 6 (RDR6)*) have reduced HR frequencies (Yao et al., 2016). Arabidopsis RAD54 mutants display reduced efficiency of somatic HR (Osakabe et al., 2006), consistent with observations that overexpression of yeast RAD54 significantly enhances HR-mediated GT in Arabidopsis seeds (Shaked et al., 2005) and egg cells (Even-Faitelson et al., 2011). However, RAD51 and RAD54 do not have the same HR-elevating effects in all plant species. In tomato, overexpression of either SlRAD51 or SlRAD54 did not enhance HR-mediated GT frequency (Van Vu et al., 2020). The different results between these two plant species suggest that further study is needed to elucidate the roles of RAD51, RAD52, and RAD54 in HR-mediated GT.

Suppression of HDR repressors has also been explored as an approach to improving HR-mediated GT. Enhanced spontaneous somatic HR frequencies were observed in Arabidopsis cells with knockout mutations in the HDR suppressors *RTEL1*, *RMI2,* and *FANCM1* (Recker et al., 2014; Röhrig et al., 2016). However, in *rtel1-1 fancm-1* and *rtel1-1 rmi2-2* Arabidopsis mutants, HR-mediated GT frequency was unaffected (Wolter and Puchta, 2019); although around 20-fold (*rtel1-1 fancm-1*) and around 80-fold (*rtel1-1 rmi2-2*) increases in HR frequency were observed in each of these double-mutants when measuring somatic HR events between the sister chromatid or homologous chromosomes (Recker et al., 2014; Röhrig et al., 2016). This discrepancy between HR and GT frequency was reasoned by Wolter and Puchta (2019) to be due to the fact that the absence of RTEL1, RMI2, and FANCM1 reduces the stability of chromosomal homologous sequences, negatively impacting DNA damage repair. Therefore, RTEL1, RMI2, and FANCM1 might not be good targets for modification for improving HR-mediated GT in plants. Modifications to other HR regulatory genes have been tested for their effects on HR frequency (Jia et al., 2012; Kwon et al., 2012; Qi et al., 2013; Steinert et al., 2016). Unlike *RTEL1*, *RMI2,* and *FANCM1*, other HR suppressors may serve as viable targets for increasing GT frequency. Improved HR frequencies were observed in rice overexpressing the DSB resection proteins OsRecQI4 and OsExo1 (Kwon et al., 2012). Additionally, in Arabidopsis, loss of the sister-chromatid-based HR-required protein SMC6B/MIM (Qi et al., 2013) and the meiotic recombination complex RAD50 and MRE11 (Gherbi et al., 2001; Jia et al., 2012) increased HR frequency. Therefore, these genes may be valuable targets for improving HR-mediated GT.

Chromatin remodeling plays a critical role in DNA repair pathways, including HR (Price and D’Andrea, 2013; Donà and Mittelsten Scheid, 2015). Altered HR frequencies have been observed in Arabidopsis chromatin-remodeling mutants. Mutations in *inositol auxotrophy 80 (INO)*, *NUCLEOSOME ASSEMBLY PROTEIN1 (NAP1)*, and the SWR1 Chromatin-Remodeling Complex subunits *ACTIN-RELATED PROTEIN6 (ARP6)* and *SWR1 COMPLEX6 (SWRC6)* result in reduced HR frequencies (Fritsch et al., 2004; Gao et al., 2012; Rosa et al., 2013). Additionally, a mutation in the nucleosome assembly gene *chromatin assembly factor 1* (*CAF-1*) was found to result in increased HR frequency (Endo et al., 2006). Given these observations, genome engineers should consider targeting chromatin-remodeling genes to improve HR-mediated GT.

Another approach to increase HDR frequency in mammalian cells is to concurrently knockout DNA polymerase theta and one of several NHEJ pathway proteins (DNA ligase IV, Ku70, and Ku80), which results in a significantly increased rate of HDR (Mateos-Gomez et al., 2015). DNA polymerase theta plays an important role in theta-mediated end joining (TMEJ), a DSB repair pathway involved in genome stability (Brambati et al., 2020). Integration of T-DNA into plant genomes was shown to be primarily DNA polymerase theta-dependent in Arabidopsis and rice (Van Kregten et al., 2016; Nishizawa-Yokoi et al., 2021). Further study showed that the mutation of Poly θ in Arabidopsis stimulates a shift from NHEJ to HDR, leading to error-prone GT elucidated by low-frequency random integration of T-DNA (Van Tol et al., 2022). Poly θ mutations in crops might provide a valuable genetic resource that may eliminate donor DNA integration into the genome and enhance the efficiency of HDR-mediated GT.

## Other Factors That Influence Plant HR Frequency

The interplay of genetic and environmental factors influences many biological processes in plants (Huang, 2016), including those that regulate HR (Boyko et al., 2005; Yao and Kovalchuk, 2011). Therefore, the effects of various environmental conditions should be considered for their role in influencing GT frequency in plants. Here, we focus on environmental factors that influence HR frequency but do not damage DNA integrity or introduce unexpected edits. These factors include temperature, light, abiotic stress, and stress-related hormone treatment.

The frequency of HR is significantly higher in Arabidopsis plants grown at either 4° or 32° C than at optimal temperatures (Boyko et al., 2005). Modulating day/night cycle also affects HR frequency. Such alterations were shown to result in over 15-fold increases in efficiency, with the lowest HR frequency observed in 24 h light/0 h dark treatment, and the greatest observed with an 8 h light/16 dark treatment (Boyko et al., 2005). These observations indicate that light and temperature altered HR efficiency in plants. Abiotic stresses such as salt, flooding, and cold stress can also increase HR frequency in Arabidopsis (Boyko et al., 2006a; Yao and Kovalchuk, 2011). Additionally, the HR frequency in plants has been demonstrated to be increased during pathogen attack (Lucht et al., 2002).

Further studies identified a potential connection between the stress-induced hormone abscisic acid (ABA) and HR (Roy and Das, 2017). Exogenous treatment of ABA enhances HR frequency, and plants with knockout mutations in the HR-related genes *AtRAD51*, *AtRAD52*, *AtRAD54,* and *AtBRCA1* display an ABA hypersensitive phenotype in seed germination (Roy and Das, 2017). Furthermore, the ABA hypersensitive mutant, *abo4-1*, exhibits a 60-fold increase in HR frequency relative to wild type (Yin et al., 2009). Although it is still largely unknown what molecular mechanisms link ABA, pathogen attack, abiotic stress, and other environmental conditions to HR frequency, genetic engineers may wish to take advantage of certain conditions to enhance HR-mediated GT efficiency. Based on the studies discussed here, growing plants in higher or lower temperatures, longer days, or treating them with ABA may result in greater GT efficiency.

Another interesting question is whether various plant cell types, tissues, or organs have unique HR frequencies. The cell types and tissues with the highest native HR frequencies would be optimal candidates for HR-mediated GT experiments. In the model dicot species Arabidopsis, the highest native HR frequencies were detected in leaves, especially on the lateral half of the leaves (Boyko et al., 2006b). However, in rice, embryogenic cells, such as callus, showed the highest HR frequencies: up to 10-fold higher than that seen in root cells and around 100-fold higher than in leaf cells (Yang et al., 2010). Notably, for the same cell type in rice, smaller sized cells demonstrated higher HR frequencies (Yang et al., 2010). Another difference between Arabidopsis and rice with regard to HR is that HR frequency in rice roots is 10 times higher than that in leaves, whereas the HR frequency in Arabidopsis roots is 2-fold lower than that in leaves, suggesting that tissue-specific HR frequency is unique in different plant species. These studies emphasize the importance of choosing the correct tissue type for GT experiments.

## Conclusion

Here, we focus on strategies that have been implemented to increase HR frequency and the efficiency of gene targeting in plants. By reviewing the advantages and disadvantages of these various approaches, tools, and methods, we provide a perspective on the potential and challenges for implementing HR in gene editing of plants. Adopting new strategies from non-plant systems, exploring novel technologies, or perhaps combining numerous strategies concurrently may further enhance our ability to precisely alter plant genomes for crop improvement and basic science research.

## Data Availability Statement

The original contributions presented in the study are included in the article/supplementary material, further inquiries can be directed to the corresponding author.

## Author Contributions

HC and MN: conceptualization, writing—original draft, and writing—review and editing. JW: writing—review and editing, supervision, and funding acquisition. All authors contributed to the article and approved the submitted version.

## Funding

This work was supported by the U.S. Department of Agriculture (grants# 2017-06529 and NCZ04214), the NC Department of Agriculture (grant# 20-070-4013), the National Science Foundation (grant# 2044721), and the National Science Foundation (grant #1940829).

## Conflict of Interest

The authors declare that the work was conducted in the absence of any commercial or financial relationships that could be construed as a potential conflict of interest.

## Publisher’s Note

All claims expressed in this article are solely those of the authors and do not necessarily represent those of their affiliated organizations, or those of the publisher, the editors and the reviewers. Any product that may be evaluated in this article, or claim that may be made by its manufacturer, is not guaranteed or endorsed by the publisher.
